# miR156a Mimic Represses the Epithelial–Mesenchymal Transition of Human Nasopharyngeal Cancer Cells by Targeting Junctional Adhesion Molecule A

**DOI:** 10.1371/journal.pone.0157686

**Published:** 2016-06-24

**Authors:** Yunhong Tian, Longmei Cai, Yunming Tian, Yinuo Tu, Huizhi Qiu, Guofeng Xie, Donglan Huang, Ronghui Zheng, Weijun Zhang

**Affiliations:** 1 Department of Radiation Oncology, Cancer Center of Guangzhou Medical University, Guangzhou, Guangdong Province, People’s Republic of China; 2 Huizhou Municipal Central Hospital, Huizhou, Guangdong Province, People’s Republic of China; University of Saarland Medical School, GERMANY

## Abstract

MicroRNAs (miRNAs) have been documented as having an important role in the development of cancer. Broccoli is very popular in large groups of the population and has anticancer properties. Junctional adhesion molecule A (JAMA) is preferentially concentrated at tight junctions and influences cell morphology and migration. Epithelial–mesenchymal transition (EMT) is a developmental program associated with cancer progression and metastasis. In this study we aimed to investigate the role of miRNAs from broccoli in human nasopharyngeal cancer (NPC). We demonstrated that a total of 84 conserved miRNAs and 184 putative novel miRNAs were found in broccoli by sequencing technology. Among these, miR156a was expressed the most. In addition, synthetic miR156a mimic inhibited the EMT of NPC cells *in vitro*. Furthermore, it was confirmed that JAMA was the target of miR156a mimic as validated by 3’ UTR luciferase reporter assays and western blotting. Knockdown of JAMA was consistent with the effects of miR156a mimic on the EMT of NPC, and the up-regulation of JAMA could partially restore EMT repressed by miR156a mimic. In conclusion, these results indicate that the miR156a mimic inhibits the EMT of NPC cells by targeting the 3’ UTR of JAMA. These miRNA profiles of broccoli provide a fundamental basis for further research. Moreover, the discovery of miR156a may have clinical implications for the treatment of patients with NPC.

## Introduction

MicroRNAs (miRNAs) are an abundant class of small non-coding RNAs that suppress gene expression at the post-transcriptional level, usually by imperfect base-pairing to the 3’ untranslated region (UTR) of a target mRNA, which results in either degradation of the mRNA or inhibition of translation [1]. miRNAs have been widely shown to modulate various cellular processes, including proliferation, differentiation, cell death, and cell mobility [2]. Furthermore, dysregulation of miRNAs has been linked to cancer and other diseases [3]. Recently, the rate of plant miRNA discoveries has increased dramatically with the advent of second-generation sequencing technology [4]. In the genus *Brassica*, 427 mature miRNAs in *Arabidopsis thaliana* have been discovered, 384 from *Arabidopsis lyrata* and 153 from *Brassica rapa* (miRBase release 20.0, June, 2013, http://mirbase.org/).

The *Brassica genus* is cultivated in most parts of the world and includes various important agronomical crops, such as *Brassica rapa*, *Brassica oleracea* var. *italica* (broccoli) and so on. Broccoli is very popular in large groups of the population owing to its flavor and its anticancer activities in prostate cancer, colon and rectal cancer, and so on [5]. Although broccoli is an important cereal crop, no miRNAs have been reported in broccoli. Interestingly, a recent report indicated that food-derived exogenous plant miR168a can pass through the gastrointestinal (GI) tract of mice and enter the circulation [6]. Therefore, we wished to investigate whether the miRNAs from broccoli had anticancer properties.

The junctional adhesion molecules A (JAMA), also known as JAM-1 and F11R, belong to a family of tight junction proteins and mediate several different physiologic processes, including intercellular junction assembly, cell polarity, and cell morphology [7,8]. Previous studies have shown that the overexpression of JAMA is a possible mechanism contributing to progression in primary breast cancer [9]. Moreover, our previous results also suggested that JAMA plays a role in regulating the epithelial–mesenchymal transition (EMT) of nasopharyngeal carcinoma (NPC), which is fairly rare in Western Europe and North America, but has a higher incidence in Southern China [10]. EMT, an early embryonic development program in which cells convert from the epithelial to the mesenchymal state, plays a pivotal role in the progression and metastasis of malignant tumors [11]. During EMT epithelial cells acquire mesenchymal features, including enhancement of motility and invasiveness, elevated resistance to apoptosis, and increased expression of mesenchymal markers such as vimentin and *N*-cadherin [12].

In this study we aimed to investigate the role of miRNAs from broccoli in NPC. Our results indicate that there are 84 conserved miRNAs and 184 novel miRNAs in broccoli. In addition, synthetic miR156a mimic, which is expressed the most in broccoli, inhibited the EMT of NPC cells *in vitro*. Furthermore, it was confirmed that JAMA was the target of miR156a mimic, as validated by 3’ UTR luciferase reporter assays and Western blotting. Knockdown of JAMA was consistent with the effects of miR156a mimic on the EMT of NPC, and the up-regulation of JAMA could partially restore EMT repressed by the miR156a mimic. Taken together, these miRNA profiles of broccoli provide a fundamental basis for further research. Moreover, our results suggest that miRNA156a mimic exhibits novel anticancer activity *in vitro*.

## Materials and Methods

### Plant Material and Growth Conditions

Seeds of broccoli (Sakata Seed Corporation, Kanagawa, Japan) were used for the experiments. The seeds were cultivated and harvested as previously described [13,14]. Small RNA Library Preparation and Sequencing

Extraction of total RNA from broccoli were performed using TRIzol reagent (Invitrogen, Carlsbad, CA, USA) according to the manufacturer’s instructions, and the samples were used as a single RNA pool. The construction of small RNA libraries and the deep-sequencing were performed by the Beijing Genomics Institute (BGI) (Shenzhen, China). Then, the analysis of small RNAs and the identification of conserved and putative novel miRNAs were performed as described in our previous study [14].

miRNA Quantification by Real-Time Reverse-Transcriptase Polymerase Chain Reaction (RT-PCR)

To validate the results from the bioinformatics-based analysis, we used stem–loop real*-*time RT-PCR. Total RNA was extracted using Trizol reagent. Then, first-strand cDNA synthesis was performed with an iScript cDNA synthesis kit (Bio-rad, Hercules, CA, USA). Real*-*time RT-PCR was carried out as previously described [14]. The reactions were repeated at least three times for sound statistical analysis. U6 were used as an internal control. The sequences of PCR primers are included in S1 Table.

### Quantification of JAMA mRNA by Real-time RT-PCR

Cells were harvested 24 hours after miR156a mimics or negative controls were transfected, and RNA was extracted using Trizol reagent following the manufacturer’s protocol. Four micrograms of total RNA was reverse transcribed into cDNA using the SuperScript First-Strand Synthesis System (Invitrogen). Real time RT-PCR was performed using SYBR Green Mix (Bio-rad). The reactions were repeated at least three times for sound statistical analysis. GAPDH expression was used as internal control. The 2^ΔΔ^*C*_T_ method has been used to calculate relative changes in gene expression determined from real-time qPCR experiment using the following equations: ^ΔΔ^*C*_T_ = (C_T, JAMA_—C_T, GAPDH_) _miRNA156a mimic_—(C_T, JAMA_—C_T, GAPDH_) _Control_, where, the *C*_T_ values were directly provided from real-time PCR instrumentation. The sequences of PCR primers are included in S1 Table.

### Cell Culture and Reagents

CNE2, HONE1 and C666-1 cell lines were available from the Cancer Institute of Southern Medical University, Guangzhou, China and were originally purchased from the American Type Culture Collection (ATCC). The three cell lines were maintained in RPMI 1640 medium supplemented with 10% fetal bovine serum (FBS, Hyclone, Logan, UT, USA) and antibiotics. MiRNA156a mimics were purchased from Ribobio (Guangzhou, China).

### Bioinformatics Analysis

Full-length cDNAs of the human genes were obtained from the National Center for Biotechnology Information (NCBI) GenBank database. A program was developed and implemented to identify miR156a-matched sites in the entire coding sequence (CDS)/UTR of the transcripts. This program used several common criteria to determine whether a transcript was a target for miR156a, as described previously [6].

### Transwell and Boyden Chamber Assays

Transwell and Boyden chamber assays were performed as described previously [15]. Cells were harvested 24 hours after miR156a mimics or negative controls were transfected, then 2 × 10^4^ cells were plated into the upper chamber and incubated for 22 hours.

### Wound Healing Assay

As described previously [15], cells transfected with miR156a mimics or negative controls were harvested and seeded into culture plates and grown under permissive conditions until they reached 90% confluence. Cells were then serum-starved for 24 hours, and similar-sized wounds were made. The distance between the wound edges was measured immediately after wounding, and again after 48 hours.

### 3’ UTR Luciferase Reporter Assay

DNA fragments of the *3’* UTR of JAMA that host the predicted complementary sites of miR156a or the mutated sites were digested by *NotI-HF* and *XhoI* (New England Biolabs, Ipswich, MA, USA). The DNA fragments were then cloned downstream of the *Renilla* luciferase reporter gene in psiCHECK2 dual luciferase reporter plasmid (Promega, Madison, WI, USA). 293T cell lines were seeded in 96-well plates and co-transfected with luciferase reporters together with miR156a mimic or negative control. Cells were lyzed with Dual-Luciferase Reporter (Promega). The luciferase activities were measured 48 hours after transfection, according to the manufacturer’s instructions, using the Panomics Luminometer (Affymetrix, Santa Clara, CA, USA). Luciferase activity was normalized by *Renilla* luciferase activity. The sequences of PCR primers used for these clonings are included in S2 Table.

### Construction of Lentivirus Vectors

The lentivirus vectors were constructed as previously described [10]. Briefly, the pWPI lentivirus vector system was obtained from Addgene (Cambridge, MA, USA). The human JAMA gene (NM_016946.4) was cloned from CNE2 cells by PCR. A JAMA fragment generated by digestion with *Pac*1 and *Pme*1 restriction enzymes was then ligated into a lineariszed pWPI plasmid. Transient transfection of cells with scramble RNAs, siRNAs targeting JAMA mRNA or miRNA156a mimics, was performed following the protocol of Lipofectamine 2000 (Invitrogen, Carlsbad, CA, USA). Sequences of PCR primers used for the constructed 3’ UTR of JAMA are included in S3 Table.

### Western Blotting Analysis

Cells were lysed in RIPA buffer. Protein mixed with loading buffer and heated at 70°C for 10 minutes were loaded on SDS-polyacrylamide gels at 30 ug per lane. The proteins were transferred to PVDF (Millipore, Massachusett, USA) after electrophoresis. Membranes were blocked for 2 hours in 5% bovine serum albumin (BSA) and incubated overnight at 4°C with the SP rabbit polyclonal antibody against E-Cadherin (Sigma), α-Catenin (Sigma), Fibronectin (Cell signaling), Vimentin (Santa Cruz), JAMA (Santa Cruz), Akt (Cell signaling), ERK (Santa Cruz) and GAPDH (Santa Cruz). Membranes were then incubated with horseradish peroxidase (HRP)-conjugated secondary antibody (1:1,000, Santa Cruz Biotechnology) for 1 hour at room temperature. Finally, bands were visualized by enhanced chemiluminescence (Thermo Scientific Pierce, Illinois, USA).

### Statistical Analysis

Statistical analysis was performed with the statistical package for social sciences (SPSS), v 15.0, and data are presented as mean ± standard deviation (SD). Statistical differences among groups were assessed with one-way analysis of variance (ANOVA). A *P*-value < 0.05 was considered statistically significant.

## Results

### miRNA Identification in Broccoli

**Fig 1:**
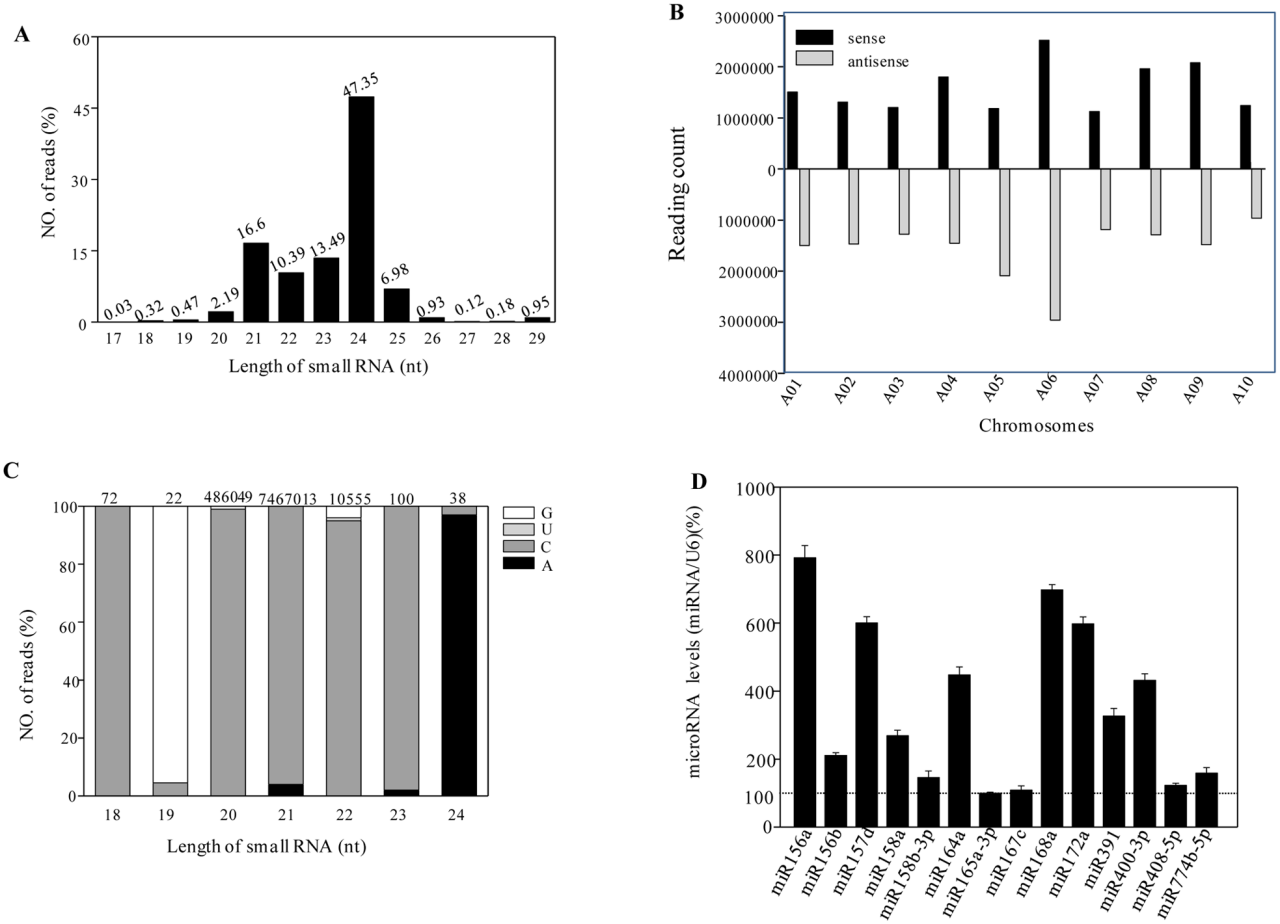
small RNAs identificatied in broccoli. (A) Length distributions of unique small RNA sequences in broccoli. Only small RNA sequences with lengths from 17 to 29 nt were considered. (B) Small RNA (redundant sequences) distribution across different chromosomes. *y* axis, number of small RNA tags located on each chromosome. *x* axis, chromosomes. Bars above the axis represent matches to the plus strand; bars below the axis represent those to the minus strand. (C) Nucleotide preferences of small RNAs. (D) The relatively high expression of conserved miRNA from Table 1 was also analyzed by real time RT-PCR.

To study the miRNAs in broccoli a small RNA library was constructed. After the removal of low-quality tags and adaptor sequences, 25 637 679 clean reads were obtained, representing 9 831 568 (38.34%) unique sequences. Thee size distribution of small RNAs was then analyzed, as shown in Fig 1A, the most abundant small RNAs were 24-nucleotide (nt) long in the broccoli library, and the second largest population was the 21-nt ones, followed by 22-nt and 23-nt small RNAs. As the genome sequence for broccoli has not yet been released, we mapped these small RNAs to the available *Brassica rapa* genome. As a result, perfect matches were obtained for about 31.2% of the total small RNAs, representing 18.1% of unique sequences. Genomic location analysis indicated that the majority of small RNAs were almost evenly located in the strand of the chromosome from A1 to A13 (Fig 1B). Moreover, most miRNA sequences start with uridine (U), whereas the majority of 24-nt siRNAs have adenosine (A) as their 5’ first nucleotide (Fig 1C).

Conserved miRNA families were found in many plant species and have important functions in plants. In our study, 84 known miRNAs were identified (Table 1). The read number of the conserved miRNAs was highly variable. For example, miR156a, miR168a, and miR157d were detected in, respectively 178 919, 118 314 and 102 632 reads, whereas the majority of broccoli miRNAs were only sequenced in < 5000 reads, and some rare miRNAs were detected in < 10 reads. Furthermore, the relatively high expression of miRNA was also confirmed by quantitative RT-PCR. Consistent with the sequencing results, miR156a, miR168a, and miR157d had the highest expression in broccoli, whereas miR165a-3p was expressed at the lowest level among the 14 miRNAs (Fig 1D). In addition, we also predicted putative new miRNA genes. Consistent with previous studies on plant miRNA and our former results, these results indicated that most miRNA sequences start with uridine (U) as their 5’ first nucleotide, and the largest population was the 21-nt miRNAs **(**Table 2). The prediction program Mireap (https://sourceforge.net/projects/mireap) was used to predict novel miRNA by exploring the secondary structure, the Dicer cleavage site, and the minimum free energy of the unannotated small RNA tags that could be mapped to the *B*. *rapa* genome. Based on these predictions, 184 miRNAs were considered novel (Table 2). With an average of 130 read counts, the putative novel miRNAs showed lower expression levels than the conserved miRNAs. These novel miRNA precursors had negative folding free energies (–18.5 to –123 kcal/mol), with an average of about –47.8 kcal/mol according to Mireap. Taken together, our results indicate that there are 84 conserved miRNAs and 184 novel miRNAs in broccoli. Moreover, miR156a was expressed the most.

**Table 1:** Conserved miRNAs and their read counts in the broccoli library.

Name	Count	Sequence
miR156a	178919	UGACAGAAGAGAGUGAGCACA
miR156b	71452	UUGACAGAAGAUAGAGAGCAC
miR157a-3p	1111	GCUCUCUAGCCUUCUGUCAUC
miR157d	102638	UGACAGAAGAUAGAGAGCAC
miR158a	47559	UUUCCAAAUGUAGACAAAGCA
miR158b-3p	47350	UUUCCAAAUGUAGACAAAGC
miR159	1315	UUUGGAUUGAAGGGAGCUCUA
miR160a	149	UGCCUGGCUCCCUGUAUGCCA
miR160a-3p	838	GCGUAUGAGGAGCCAUGCAUA
miR161	105	UCAAUGCACUGAAAGUGACUA
miR162a	1064	UCGAUAAACCUCUGCAUCCAG
miR164a	5886	UGGAGAAGCAGGGCACGUGCA
miR165a-3p	36135	UCGGACCAGGCUUCAUCC
miR167a-3p	48	GAUCAUGUUCGUAGUUUCACC
miR167c	68502	UGAAGCUGCCAGCAUGAUCUA
miR168a	118314	UCGCUUGGUGCAGGUCGGGAA
miR168a-3p	424	CCCGCCUUGCAUCAACUGAAU
miR169d-3p	139	GCAAGUAGACUUUGGCUCUGU
miR169m	1207	UGAGCCAAAGAUGACUUGCCG
miR171f	1020	UGAUUGAGCCGCGCCAAUAUC
miR172a	29687	AGAAUCUUGAUGAUGCUGCAU
miR173-5p	11	UUCGCUUGCAGAGAGAAAUCAC
miR1885b	5580	UACAUCUUCUCCGCGGAAGCUC
miR2111c	18	UAAUCUGCAUCCUGGGGUUUA
miR319b-5p	2913	GAGCUUUCUUCGGUCCACUC
miR3440b-3p	1429	GGGGAUUGGUCAAGGGAAGAU
miR3447-3p	110	UAUGAGUCUGUUUGUUGAUGACA
miR390a	1445	AAGCUCAGGAGGGAUAGCGCC
miR390a-3p	36	CGCUGUCCAUCCUGAGUUUCA
miR391	5776	UUCGCAGGAGAGAUAGCGCCA
miR391-3p	70	ACGGUAUCUCUCCUACGUAGC
miR393	179	UCCAAAGGGAUCGCAUUUUU
miR393b-3p	17	AUCAUGCGAUCUCAUUGGAUU
miR394a	16	UUGGCAUUCUGUCCACCUCC
miR396a	11	UUCCACAGCUUUCUUGAACUG
miR396b-3p	98	CUCAAGAAAGCUGUGGGAAA
miR397a	219	UCAUUGAGUGCAGCGUUGAUGU
miR398a-5p	20	AGAGGUGCAUGAGAACAGGGA
miR399b	88	UGCCAAAGGAGAGUUGCCCUG
miR400	38	UAUGAGAGUAUUAUAAGUCAC
miR400-3p	10669	GACUUAUAAUAAGUCGUCUGGA
miR403	1541	UUAGAUUCACGCACAAACUCG
miR408-5p	44493	ACAGGGAACAAGCAGAGCAUG
miR415	24	AACAGAGACAGUAAAACAGAGACA
miR4249	60	UGAAUUAGUAGAAUUUGAGGA
miR472	48	UUUUGCCUACUCCUCCCAUACC
miR5021	627	UUAGAAGAAGAAGAAGAACAUA
miR5024-5p	8	AUUGACAAGGACAAAUAUCAACAA
miR5026	20	ACUCAUAAGAUCGUGACACGU
miR5633	397	UUUGAUCAUCAGAAAGACAUA
miR5634	56	AGGAGAUUUGUGAGCAUUUAGCGG
miR5638b	114	ACAGUGGCAUCAUAGGUGGGGCUG
miR5641	131	AGGAAGAAGAUGAAACUAGAAUUA
miR5654a	1474	AUAAAUCCCAAGCAUCAUCCA
miR5655	69	AGAGAUUAGAACACAUAAAAGGAG
miR5658	918	AUUGGAUGAUGAUGAAGAUGACA
miR5661	9	AUGAGGUACAUCAUGUAGUCUGAA
miR5711	541	UGUUCUGUGGGUUUUUACCGA
miR5716	31	UUGGAUAAGUGAAGAUAUAAA
miR5718	2514	UCAGAACCAAACCCAGAACAAG
miR5719	338	UUGUGAUGAUAAUCCGACUCCAU
miR5720	48	UUUGUGAUUUGGUUGGAAUAUC
miR5721	290	AGAAAAUGGAGUGAAAAUGAG
miR6028	39	UGGAGAGUAAAUGACAUUCAGA
miR6030	4193	UCCACCCAUACCAUACAGACCC
miR6031	1652	AUGAGAUUCGGAGCGGUUUGAAGC
miR6034	1584	UCUGAUGUAUAUAGCUUUGGG
miR6035	536	AUGGAGUGGAAAAUGCAGUCAUA
miR774b-5p	9834	UGAGAUGAAAGAUCUUGGACAGAC
miR824	1210	UAGACCAUUUGUGAGAAGGGA
miR824-3p	136	CCUUCUCAUCGAUGGUCUAGA
miR827	1055	UUAGAUGACCAUCAACAAAUA
miR829.1	769	ACAGCUCUGAUACCACAUGAUGGA
miR831-5p	1771	AGAUGAGGACGAGGAAGAUGAUGA
miR837-3p	1579	AAACGAACAAGAAUUGACGCA
miR841	43	AUACAACCCACUGGAAACUGAUA
miR845a	232	UUUGGCUCUGAUACCAAUUGAUGU
miR845b-3p	206	GUUUGCUCUGAUACCAAAUGAUGG
miR847-5p	83	UCUGAUGAAGAGGAAUGGUCAA
miR855	155	AGAAAAGCUGAAGGAUAAGUGCAA
miR858-3p	262	UCGAACAGACAACGAAGAUU
miR860	30	UCAAUACAUUGGACUACAUAU
miR861-3p	213	UGAUGGAUAUGUACCUAUCAAGGA
miR867	45	UGUGAACGUGGUUGAUUAGGAUA

**Table 2:** Putative novel miRNAs and their read counts in the broccoli library.

ID	MFE	Id(5p)	ID (3p)	Count	Count(5p)	Count(3p)	Sequence (5p)	Sequence (3p)
Bro-m0001	-38.3	Bro-m0001-5p	-	167	166	-	TGAGTCTGTATAAGTTGATGACA	-
Bro-m0002	-40.1	Bro-m0002-5p	-	49	48	-	TACACTAGTTGTGGACATGGACA	-
Bro-m0003	-70.8	Bro-m0003-5p	-	697	664	-	TTGTGCACATGTGGATAGGCTTA	-
Bro-m0004	-56.1	Bro-m0004-5p	Bro-m0004-3p	34	17	16	AAGGGCGGGTCAAAATCTAGTTA	AACTAGATTTTGATCCGCGCTTA
Bro-m0005	-78.7	Bro-m0005-5p	-	22	22	-	TGAAGGAATAGAGAGTGGAAT	-
Bro-m0006	-45.9	Bro-m0006-5p	-	336	333	-	AGAGCTGTTGGCGAGATTCCTA	-
Bro-m0007	-29.9	Bro-m0007-5p	-	92	92	-	GCGGTTTGAAGCGGTTTAGAACA	-
Bro-m0008	-76.9	-	Bro-m0008-3p	18	-	18	-	TCGTTACGGATCGGTCGCTAC
Bro-m0009	-41.3	-	Bro-m0009-3p	36	-	35	-	TGGACTTGTTTGATGGGCAGCTA
Bro-m0010	-24.4	-	Bro-m0010-3p	23	-	23	-	AGAAGACGACAAGAGCTCAAGTT
Bro-m0011	-32.3	Bro-m0011-5p	-	329	314	-	TTGAAGCGATTTGTAGCGGTTTA	-
Bro-m0012	-30.9	Bro-m0012-5p	-	40	40	-	GTCGATCGACGGCGAAGCGCGCA	-
Bro-m0013	-89.9	-	Bro-m0013-3p	25	-	25	-	TTAGTTTCGGTTCGGTTCGGTTT
Bro-m0014	-90.3	-	Bro-m0014-3p	25	-	24	-	TACACATATGAATCTTTAGAA
Bro-m0015	-43.7	Bro-m0015-5p	-	263	260	-	TACAATGCTGGAGCGTCGCACA	-
Bro-m0016	-24.9	-	Bro-m0016-3p	62	-	61	-	TCGGACGTTTGTGACGGATTTCC
Bro-m0017	-23.91	Bro-m0017-5p	-	45	45	-	GTCGATCGACGGCGAAGCGCGCA	-
Bro-m0018	-23	-	Bro-m0018-3p	29	-	28	-	AAACCAGGATGAATCGCGATGTA
Bro-m0019	-66.5	Bro-m0019-5p	-	90	89	-	AATGGAGTGGGAAATGGAGTA	-
Bro-m0020	-77.7	Bro-m0020-5p	-	90	89	-	AATGGAGTGGGAAATGGAGTA	-
Bro-m0021	-40.2	Bro-m0021-5p	-	246	246	-	GCAGCACCATCAAGATTCACA	-
Bro-m0022	-18.7	-	Bro-m0022-3p	48	-	48	-	ATATGTAGAGATTTTTGTTACTA
Bro-m0023	-73.7	-	Bro-m0023-3p	86	-	83	-	TTGCGGACGGTTGCGGGAGGATA
Bro-m0024	-51.8	Bro-m0024-5p	Bro-m0024-3p	20	1	18	ACCTAGATGACTTACATGGAAGT	TTCCATGTAAGTCGTCTAGGATA
Bro-m0025	-75.5	Bro-m0025-5p	-	90	89	-	AATGGAGTGGGAAATGGAGTA	-
Bro-m0026	-38	Bro-m0026-5p	-	20	20	-	GAAGATGTTGGAAGCGGTGATCA	-
Bro-m0027	-52.8	-	Bro-m0027-3p	24	-	24	-	TTAGTGTTTTGCTGAAGACGTTA
Bro-m0028	-27.3	-	Bro-m0028-3p	44	-	44	-	ATACTAGATCTCGATCCGCGC
Bro-m0029	-79.4	Bro-m0029-5p	-	38	38	-	AAGCTGGTTGGCAGGTACACTCA	-
Bro-m0030	-43.7	Bro-m0030-5p	-	20	20	-	TGGAGTAGTAGAACACGTGCG	-
Bro-m0031	-28.9	-	Bro-m0031-3p	21	-	21	-	TCTGTCGCGAAGCTTGGCCACTC
Bro-m0032	-43.8	Bro-m0032-5p	Bro-m0032-3p	830	32	797	ATCTGCCGACTCATCCATCCA	TGTGAATGATGCGGGAGATGT
Bro-m0033	-78.3	Bro-m0033-5p	-	90	89	-	AATGGAGTGGGAAATGGAGTA	-
Bro-m0034	-35.7	Bro-m0034-5p	-	19	19	-	AAGGGCGGGTCAAAATCTAGTTA	-
Bro-m0035	-48.4	Bro-m0035-5p	-	56	55	-	TGGAGGCAGCGGTTCATCGAT	-
Bro-m0036	-49.5	-	Bro-m0036-3p	555	-	553	-	GCGTACAGAGTAGTCAAGCATG
Bro-m0037	-62.5	Bro-m0037-5p	Bro-m0037-3p	24	2	22	CTCGGATTCGCTTGGTGCAGG	TGCATCAACTGAATCGGAGCC
Bro-m0038	-78.4	Bro-m0038-5p	-	90	89	-	AATGGAGTGGGAAATGGAGTA	-
Bro-m0039	-47.1	Bro-m0039-5p	-	22	22	-	AGGATATTTTGCTGATTTGTCAT	-
Bro-m0040	-85.7	Bro-m0040-5p	Bro-m0040-3p	161	14	145	GGAGCTTCCTTTAGTCCATTC	TTGGACTGAAGGGAGCTCCCT
Bro-m0041	-45.5	Bro-m0041-5p	-	246	246	-	GCAGCACCATCAAGATTCACA	-
Bro-m0042	-24.1	-	Bro-m0042-3p	19	-	19	-	TGAATGGAATGGAATACTCAT
Bro-m0043	-38.8	Bro-m0043-5p	-	32	31	-	TTTTTAGCGGAATATAAGAAT	-
Bro-m0044	-22.2	Bro-m0044-5p	-	207	200	-	CGTGATCTTCGTAGGACGGCTCA	-
Bro-m0045	-80.4	Bro-m0045-5p	-	90	89	-	AATGGAGTGGGAAATGGAGTA	-
Bro-m0046	-43.2	Bro-m0046-5p	-	96	96	-	TTAGGGTTTAGAGTTAAAGGGTA	-
Bro-m0047	-21.9	-	Bro-m0047-3p	22	-	21	-	TTGCGGGGTACAAAGACGTTTG
Bro-m0048	-34.4	-	Bro-m0048-3p	23	-	23	-	CGGTGGACGGTCATTAGGCGCTA
Bro-m0049	-60.7	-	Bro-m0049-3p	17	-	16	-	AAGAGACCGGAGTTAATCAT
Bro-m0050	-61.7	Bro-m0050-5p	-	90	89	-	AATGGAGTGGGAAATGGAGTA	-
Bro-m0051	-47.3	Bro-m0051-5p	-	479	471	-	GTTCGGACGCGGCGTCAGACACA	-
Bro-m0052	-38.3	-	Bro-m0052-3p	39	-	38	-	AACGAACAACAACCTGCGACTTA
Bro-m0053	-39.2	Bro-m0053-5p	Bro-m0053-3p	832	31	797	ATCTGCCGACTCATCCATCCA	TGTGAATGATGCGGGAGATGT
Bro-m0054	-25.9	-	Bro-m0054-3p	90	-	89	-	AATGGAGTGGGAAATGGAGTA
Bro-m0055	-61	Bro-m0055-5p	-	35	35	-	CCAGTAGTTCGTTAGGTTTGAC	-
Bro-m0056	-56.5	Bro-m0056-5p	-	85	83	-	GGAATGTTGTTTGGCTCGAAG	-
Bro-m0057	-59	Bro-m0057-5p	-	89	88	-	AATGGAGTGGGAAATGGAGTA	-
Bro-m0058	-29.2	Bro-m0058-5p	-	46	46	-	GGAGACACAAAAAACGAGACGCC	-
Bro-m0059	-67.1	-	Bro-m0059-3p	18	-	18	-	TCGACAACCGACAATACTACCTA
Bro-m0060	-45.3	-	Bro-m0060-3p	116	-	116	-	TTTGTTCATGACTGCATTTTC
Bro-m0061	-51.7	Bro-m0061-5p	-	38	38	-	CGGTCTGTGATGGTACTAGTA	-
Bro-m0062	-45.1	Bro-m0062-5p	Bro-m0062-3p	1051	253	797	ATCTGCCGACTCATTCATCCA	TGTGAATGATGCGGGAGATGT
Bro-m0063	-27.6	Bro-m0063-5p	-	23	22	-	TTAGTTTCGGTTCGGTTCGGTTT	-
Bro-m0064	-27.6	-	Bro-m0064-3p	29	-	28	-	TTTTCAGATCTGGAAGACTTCTT
Bro-m0065	-51.5	Bro-m0065-5p	-	19	18	-	CGGCAGTGTTTCGTCGGTCGATA	-
Bro-m0066	-89.5	-	Bro-m0066-3p	26	-	26	-	TTGCGACATTGAGAAGACTTTC
Bro-m0067	-36.6	Bro-m0067-5p	-	2669	2665	-	TGGACGACTTAGTAGATGACTT	-
Bro-m0068	-108	Bro-m0068-5p	Bro-m0068-3p	50	49	1	ATATGTAGAGATTTTTGTTACTA	GTAACAAAAATCTCTACATATAC
Bro-m0069	-56.4	Bro-m0069-5p	-	58	58	-	TCCGAATGCAAGGCTGATTCGC	-
Bro-m0070	-48.2	-	Bro-m0070-3p	104	-	104	-	TGAATGTCTTTCTCTTCATC
Bro-m0071	-40.5	Bro-m0071-5p	-	38	37	-	AATTGTTTGATCGTCTTTCTC	-
Bro-m0072	-80.1	Bro-m0072-5p	-	91	89	-	AATGGAGTGGGAAATGGAGTA	-
Bro-m0073	-43	-	Bro-m0073-3p	289	-	288	-	ATGCACTGCCTCTTCCCTGGC
Bro-m0074	-59.6	Bro-m0074-5p	-	377	376	-	GGAATGTTGTCTGGCTCGAGG	-
Bro-m0075	-40	Bro-m0075-5p	-	21	20	-	TCTGGATCTGGATGAGTTCGGTT	-
Bro-m0076	-69.7	Bro-m0076-5p	-	62	60	-	TGGAAGACTTTCCAGACGACTT	-
Bro-m0077	-24.4	Bro-m0077-5p	-	34	34	-	AGAAGATCTATATTTAAGAACTA	-
Bro-m0078	-24.1	-	Bro-m0078-3p	19	-	18	-	ACTGTTAACTGCGGTGCGGAG
Bro-m0079	-29.5	Bro-m0079-5p	-	629	619	-	TTTGGTTGGGCTTCAAGGCTG	-
Bro-m0080	-40.4	Bro-m0080-5p	-	294	294	-	GGAATGTTGTCTGGCTCGAGG	-
Bro-m0081	-61.6	Bro-m0081-5p	Bro-m0081-3p	74	1	72	AGCCAAGGATGACTTGCCGGA	TGGCAAGTTGTCCTTCGGCTAC
Bro-m0082	-44.8	Bro-m0082-5p	-	19	19	-	TGGAGTAGTAGAACACGTGCG	-
Bro-m0083	-122.8	Bro-m0083-5p	-	374	373	-	CAACAGTCGACGGGTTCGGAA	-
Bro-m0084	-30.6	-	Bro-m0084-3p	41	-	41	-	TTTGACTTTTCCTAGACGACTT
Bro-m0085	-41.4	Bro-m0085-5p	-	19	18	-	TTAGCGTAATTTAAGAACCGGTT	-
Bro-m0086	-47.3	-	Bro-m0086-3p	109	-	109	-	GCGTACAAGGAGTCAAGCATG
Bro-m0087	-38.7	-	Bro-m0087-3p	138	-	138	-	AATGGAGTGGGAAATGGAGTA
Bro-m0088	-47.7	-	Bro-m0088-3p	113	-	113	-	AGATATTAGTGCGGTTCAATC
Bro-m0089	-46	-	Bro-m0089-3p	132	-	131	-	AATGGAGTGGGAAATGGAGTA
Bro-m0090	-33.9	-	Bro-m0090-3p	32	-	32	-	AGAGACGTCTCTTAGCTTTTT
Bro-m0091	-62.3	-	Bro-m0091-3p	21	-	20	-	AGAAGAAACGTAGAAAACACTT
Bro-m0092	-37.8	Bro-m0092-5p	-	679	647	-	TTTGTTTAAGTTCTGTGTCGGTA	-
Bro-m0093	-44.8	Bro-m0093-5p	-	22	22	-	TTACAAGAGATCTGCAGCATG	-
Bro-m0094	-32.4	-	Bro-m0094-3p	29	-	29	-	AGACCAAGCTACTCACACTAACA
Bro-m0095	-35.7	-	Bro-m0095-3p	25	-	25	-	TCTGGAGGAGTTGATAGACCAT
Bro-m0096	-70.4	Bro-m0096-5p	-	92	91	-	AATGGAGTGGGAAATGGAGTA	-
Bro-m0097	-84.1	Bro-m0097-5p	-	67	67	-	ATATACTCTCAAGCATATCAA	-
Bro-m0098	-98.5	-	Bro-m0098-3p	21	-	20	-	AAGCACGATTTTGAGAGTTCAT
Bro-m0099	-32.1	-	Bro-m0099-3p	85	-	84	-	AGCTTTTGCGGTTTGTAGCGGTT
Bro-m0100	-19.7	Bro-m0100-5p	-	20	19	-	TTAAACAAAGTGAGAAGTAGACA	-
Bro-m0101	-38.8	-	Bro-m0101-3p	35	-	34	-	AGTAGACGACTTATTTTTCA
Bro-m0102	-69.3	Bro-m0102-5p	Bro-m0102-3p	32	30	2	TTCCATGTAAGTCGTCTAGGATA	TTTGGACGACTTACATGGAAGT
Bro-m0103	-90.4	Bro-m0103-5p	-	67	67	-	ATATACTCTCAAGCATATCAA	-
Bro-m0104	-55.2	Bro-m0104-5p	-	19	18	-	TCTTCGACACTACTTAAGACTTA	-
Bro-m0105	-40.8	Bro-m0105-5p	Bro-m0105-3p	19	4	15	ATCACAAAAATAACACTCAAAAAC	CTTTGAGTGCTAGTTTGGGAACA
Bro-m0106	-60.3	-	Bro-m0106-3p	20	-	20	-	TATGGGTGTTGAATTAATAGGTA
Bro-m0107	-71.8	-	Bro-m0107-3p	30	-	30	-	TTCCCACAAGAACGAAAACTC
Bro-m0108	-22.8	-	Bro-m0108-3p	30	-	30	-	AAAACAAAGTATGAATGGTGACA
Bro-m0109	-25.2	Bro-m0109-5p	-	45	45	-	TGGCGATGACGAAGAAAAAA	-
Bro-m0110	-59.2	Bro-m0110-5p	-	22	21	-	CGGAATTCCGTCGGAATATTCCA	-
Bro-m0111	-76.1	Bro-m0111-5p	-	22	22	-	AATGGGCCTTGAGGGAGGATGTA	-
Bro-m0112	-29.2	Bro-m0112-5p	-	46	46	-	GGAGACACAAAAAACGAGACGCC	-
Bro-m0113	-43	Bro-m0113-5p	-	23	22	-	AGGATATTTTGCTGATTTGTCAT	-
Bro-m0114	-38.6	Bro-m0114-5p	-	22	22	-	TTTGTCAGTTTTCGTGTGTGACA	-
Bro-m0115	-39	Bro-m0115-5p	-	113	113	-	AGATATTAGTGCGGTTCAATC	-
Bro-m0116	-58.4	Bro-m0116-5p	Bro-m0116-3p	23	7	16	ACAGGTTCATGAGTGTTTTGG	TAATATTTATGTGCTCTGTCA
Bro-m0117	-57.2	-	Bro-m0117-3p	18	-	18	-	CCAAGCCACTCGGTCGGTCGCTA
Bro-m0118	-39	-	Bro-m0118-3p	33	-	32	-	GGAAATGAAGATTTGTTGGTTTA
Bro-m0119	-28.9	-	Bro-m0119-3p	21	-	21	-	TCTGTCGCGAAGCTTGGCCACTC
Bro-m0120	-73.2	Bro-m0120-5p	-	17	16	-	TACTCAAGTATTATATGCGCA	-
Bro-m0121	-35.1	Bro-m0121-5p	Bro-m0121-3p	29	28	1	TAGCTCCAGACTCATTCACTCA	AGTAAATGATGCGGGAGACTTAT
Bro-m0122	-29.8	-	Bro-m0122-3p	22	-	22	-	TGATGATGATGAAGCTGATGA
Bro-m0123	-34.4	Bro-m0123-5p	-	77	76	-	TCGGATAAGAGACGGTTCTT	-
Bro-m0124	-35.2	-	Bro-m0124-3p	60	-	58	-	GGCAGACTAAGGTAAAAAGGACA
Bro-m0125	-44.7	Bro-m0125-5p	-	95	93	-	GGATAGTTAGGAATGTGGCTG	-
Bro-m0126	-37.1	Bro-m0126-5p	-	31	31	-	TGGAGTAGTAGAACACGTGCA	-
Bro-m0127	-76.4	Bro-m0127-5p	Bro-m0127-3p	316	144	165	TGGATGCATAACCAGTAGATA	TATCTACTGCTTATGCCACCA
Bro-m0128	-40.2	Bro-m0128-5p	Bro-m0128-3p	108	3	104	AGTCTTCTCGGATAAATATGTTAGTT	TTGACTTTTTGTAGAAGACTTC
Bro-m0129	-92.6	Bro-m0129-5p	-	25	24	-	TTGGAACAAGAAGATGAAGAATA	-
Bro-m0130	-19.2	-	Bro-m0130-3p	32	-	31	-	ATTCTAAGTCGTCGTAGATGGTA
Bro-m0131	-49.6	-	Bro-m0131-3p	50	-	50	-	GCGTACGAGGAGCCAAGCATG
Bro-m0132	-72.2	-	Bro-m0132-3p	81	-	78	-	CGGAGCCATGCGAAGCGACACA
Bro-m0133	-23.1	Bro-m0133-5p	Bro-m0133-3p	18	9	8	AGTCTAGCTCAGTTCGGATATTT	GTATCCGAAAAGACGAAAGACGAA
Bro-m0134	-18.7	Bro-m0134-5p	Bro-m0134-3p	100	97	1	AATCAACACTGTGGGCTGAGGCA	CCGTCAATTCCAGGAAGATGGT
Bro-m0135	-49.1	Bro-m0135-5p	-	23	23	-	TCCGACGACTTTTCGACGACAT	-
Bro-m0136	-27.3	-	Bro-m0136-3p	63	-	61	-	TTCTTATCCGTAGAATACAGCTA
Bro-m0137	-114.6	-	Bro-m0377-3p	18	-	17	-	GATTTTTACGGATTTTCAGACA
Bro-m0138	-49.1	Bro-m0138-5p	-	17	17	-	GCATCATCATCAAGATTCAGA	-
Bro-m0139	-48.9	Bro-m0139-5p	-	29	28	-	GGACTGTTGTCTGGCTCGAGG	-
Bro-m0140	-39.7	Bro-m0140-5p	-	114	113	-	AGATATTAGTGCGGTTCAATC	-
Bro-m0141	-23	Bro-m0141-5p	-	18	18	-	AAGAGACCGGAGTTAATCAT	-
Bro-m0142	-56.2	Bro-m0142-5p	-	253	249	-	AGAGCTTTGGAACAGACTGCA	-
Bro-m0143	-19.2	Bro-m0143-5p	-	17	17	-	TGAGAGGGTTGTATACGTCGTTA	-
Bro-m0144	-28.9	-	Bro-m0144-3p	21	-	21	-	TCTGTCGCGAAGCTTGGCCACTC
Bro-m0145	-71.5	Bro-m0145-5p	-	69	67	-	TCAGCAGACAGGTCAGAAATTCA	-
Bro-m0146	-21.8	Bro-m0146-5p	-	86	84	-	AAACAACCGTCGTTTGTCGTTCA	-
Bro-m0147	-35.3	Bro-m0147-5p	-	20	20	-	AGGCTAAAACATCTGATTGTCTA	-
Bro-m0148	-114.6	-	Bro-m0148-3p	18	-	17	-	GATTTTTACGGATTTTCAGACA
Bro-m0149	-30.4	Bro-m0149-5p	-	95	92	-	GCGGTTTGAAGCGGTTTAGAACA	-
Bro-m0150	-67.1	Bro-m0150-5p	-	48	48	-	TGGGCTGGTTTTGGGGTGTTACA	-
Bro-m0151	-33.9	Bro-m0151-5p	-	18	17	-	TTTGTTAAAGTTAAGAACCGGTT	-
Bro-m0152	-44.7	-	Bro-m0152-3p	17	-	17	-	ACTAGCGGAATGTGTCGGGTTTA
Bro-m0153	-46.7	Bro-m0153-5p	-	189	188	-	TTGTGCAAGACTAAGAAGCAA	-
Bro-m0154	-76	-	Bro-m0154-3p	62	-	61	-	AGGCTAGTGATGGATTTAGAA
Bro-m0155	-18.5	-	Bro-m0155-3p	697	-	664	-	TTGTGCACATGTGGATAGGCTTA
Bro-m0156	-42.1	Bro-m0156-5p	Bro-m0156-3p	72	64	7	TGAGCAGCTGGAGTTCATAGAT	CTGTGAATCGAGTCTGTTTAAG
Bro-m0157	-44.5	-	Bro-m0157-3p	39	-	39	-	TCTGATACCAACTGATGTAGCGA
Bro-m0158	-45.3	Bro-m0158-5p	-	252	252	-	GCAGCACCATTAAGATTCACA	-
Bro-m0159	-81.7	-	Bro-m0159-3p	21	-	20	-	GCGGGACGAACGCTCGGTCGCTA
Bro-m0160	-48.6	Bro-m0160-5p	-	40	40	-	GGAATGTTGTCTGGATCGAGGA	-
Bro-m0161	-22.17	Bro-m0161-5p	-	47	47	-	AAAGGAAAGTTAGAAGACTTCTA	-
Bro-m0162	-48.2	Bro-m0162-5p	-	142	141	-	AAGCAACCGTCTTTCGTCGTTCA	-
Bro-m0163	-77.1	-	Bro-m0163	18	-	18	-	TACGGAAGTTTTGAAGATAATTA
Bro-m0164	-38.1	Bro-m0164-5p	-	37	36	-	CTTATAATTAAGTCGTCTGG	-
Bro-m0165	-27.4	Bro-m0165-5p	-	45	44	-	CTGATTAGGTAGCGTAGGATCCA	-
Bro-m0166	-34.7	-	Bro-m0166-3p	18	-	18	-	TTCTTTGTAAGTCTTCTCGTTCA
Bro-m0167	-26.6	Bro-m0167-5p	-	28	27	-	AGACAAGACAGAGAGTTAGTTCA	-
Bro-m0168	-26.1	Bro-m0168-5p	-	129	128	-	CGGGATTTAACGGAATAGCCC	-
Bro-m0169	-60.6	-	Bro-m0169-3p	33	-	33	-	TTTAGAACTCCAATGGGTAGA
Bro-m0170	-58	-	Bro-m0170-3p	1286	-	1276	-	AAGACTTTCCAGACGACTTAA
Bro-m0171	-62.1	Bro-m0171-5p	-	18	17	-	TGAGAGGGTTGTATACGTCGTTA	-
Bro-m0172	-67	-	Bro-m0172-3p	18	-	17	-	TGAGAGGGTTGTATACGTCGTTA
Bro-m0173	-49	Bro-m0173-5p	Bro-m0173-3p	21	14	7	AAGCTCGGTCGCTACGTAGCA	CTACGTAGCGACCGAGCGTCC
Bro-m0174	-25.3	Bro-m0174-5p	-	17	17	-	TGAGAGGGTTGTATACGTCGTTA	-
Bro-m0175	-32.4	Bro-m0175-5p	-	348	343	-	AGAGCTGTTGGCGAGATTCCTA	-
Bro-m0176	-40.2	Bro-m0176-5p	Bro-m0176-3p	23	19	3	GGAGCTAGTCAGATATGCGGA	TGCATGGTGGATGGTTCATG
Bro-m0177	-28.9	-	Bro-m0177-3p	21	-	21	-	TCTGTCGCGAAGCTTGGCCACTC
Bro-m0178	-36.8	-	Bro-m0178-3p	17	-	16	-	TCCGATGGCTTATGTTTGATCTA
Bro-m0179	-39.7	-	Bro-m0179-3p	32	-	32	-	AAGGAGACAAGATCAAAGGTTTA
Bro-m0180	-76.9	Bro-m0180-5p	Bro-m0180-3p	1356	1291	15	TGGACGACCTTCTAGACGACTT	CTTACTTGAAGGTCGTCCACG
Bro-m0181	-88.7	Bro-m0181-5p	Bro-m0181-3p	24	4	19	GCGACTGAGCGGGACGAGCGCTC	GCGGGACGAACGCTCGGTCGCTA
Bro-m0182	-26.4	Bro-m0182-5p	-	124	123	-	AATCACTTGGAGATTGTCGAACA	-
Bro-m0183	-21.7	-	Bro-m0183-3p	335	-	333	-	AGAGCTGTTGGCGAGATTCCTA
Bro-m0184	-60.7	-	Bro-m0184-3p	1408	-	1370	-	TTTGACTAGCGGGTTGACTTTT

### MiR156a Mimics Repress the EMT of NPC Cells *In Vitro*

**Fig 2:**
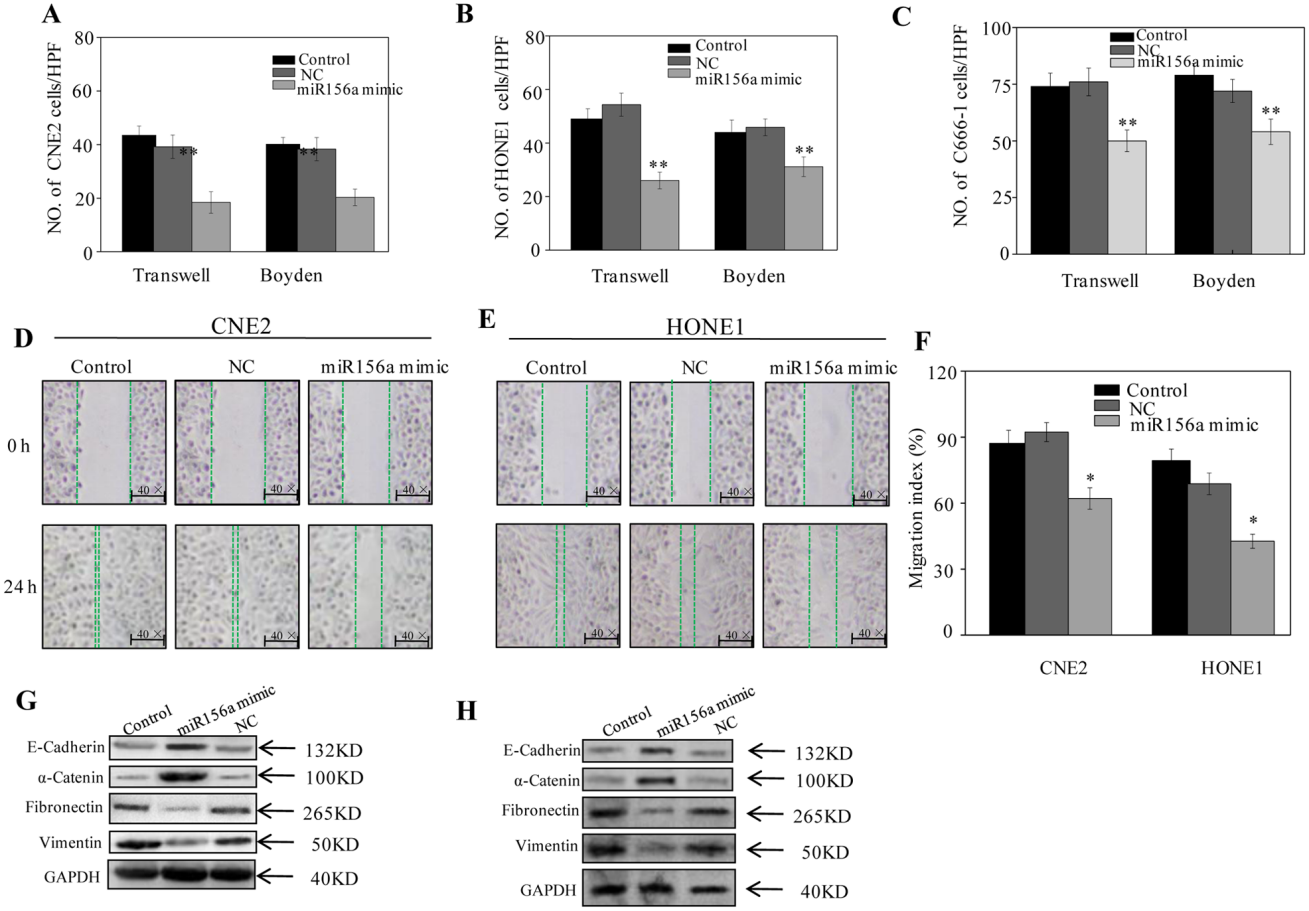
Broccoli miR156a mimic represses the EMT phenotype of NPC cells *in vitro*. Cells are harvested 24 hours after transfection. (A) The invasive properties of the CNE2 (A), HONE1 (B) and C666-1 (C) cells were measured by transwell and Boyden chamber assays. A wound-healing assay of CNE2 (D) and HONE1 (E) cells was performed. The total distance migrated by wounded cells is expressed as a percentage of the initial distance (F). (C) The expressions of epithelial (E-cadherin and α-catenin) and mesenchymal markers (vimentin and Fibronectin) in CNE2 (G) and C666-1 (H) were measured by western blotting. Data are presented as mean ± SD (n = 3). Significant differences between the negative controls and miRNA156a mimics indicated by *P < 0.05 and **P < 0.01.

To address the question whether miR156a from broccoli exhibits anticancer activity, we conducted an analysis of miR156a mimic on the EMT of NPC *in vitro*. The cellular migration and invasive activities of NPC cells upon stimulation with miR156a mimic were investigated by transwell and Boyden assays. We found that miR156a mimic significantly suppressed the invasion of CNE2, HONE1 and C666-1 cells in Boyden assays and reduced migration in transwell assays (Fig 2A, 2B and 2C). Moreover, the migration index decreased significantly in CNE2 and HONE1 cells when miR156a mimics were transfected (Fig 2D, 2E and 2F). In miR156a mimic-transfected cell lines, western blotting revealed an increase in expression levels of α-catenin and E-cadherin, and a decrease in expression levels of the mesenchymal cell markers fibronectin and vimentin (Fig 2G and 2H). These results indicate that miR156a mimic represses the EMT of NPC cells *in vitro*.

### miR156a Mimic Directly Targets the 3’ UTR of JAMA and Reduces JAMA Protein Levels *In Vitro*

**Fig 3:**
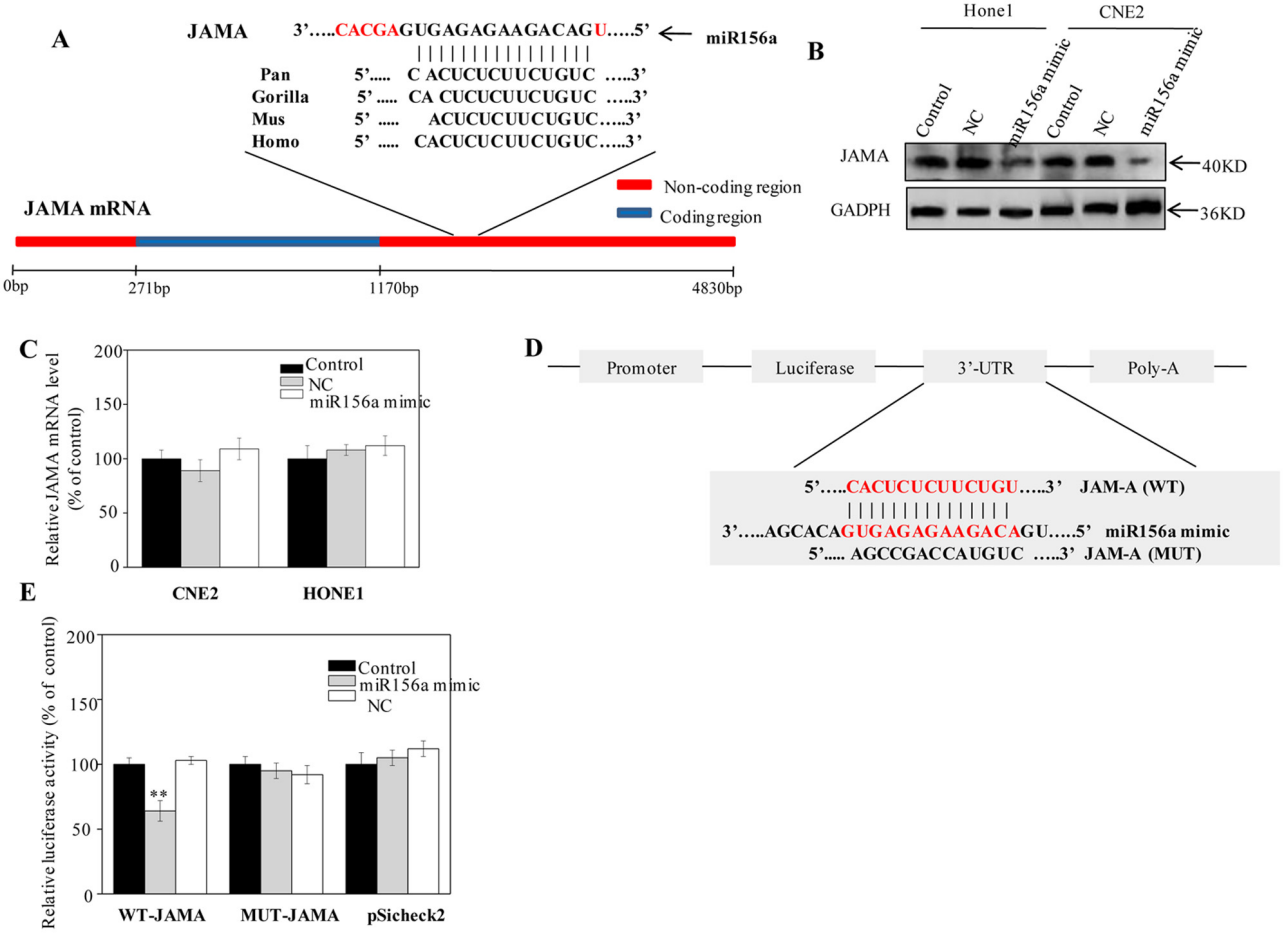
miR-156a mimic directly regulates JAMA by binding to the 3’ UTR of JAMA. (A) Schematic description of the hypothesized duplexes formed by interactions between the 3’ UTR and miR156a. Note that the potential binding site of MIR156a to JAMA mRNA is highly conserved across species. (B) Western blotting analysis of JAMA protein levels in miR168a-transfected NPC cells. (C) Real-time RT-PCR analysis of JAMA mRNA levels in miR168a-transfected NPC cells. (D) Diagram of the luciferase reporter plasmid carrying the firefly luciferase-coding sequence attached to the wild type or mutant miR156a complementary site. (E) Luciferase activities in 293T cells co-transfected with luciferase reporters and miR156a or NC (n = 6). *P < 0.05; **P < 0.01 compared with negative controls.

To understand the underlying molecular mechanism by which miR156a suppresses NPC cell invasion and metastasis, we searched for miR156a targets using different computational methods according to a previous study [6] and the basic local alignment search tool (BLAST) of NCBI (http://blast.ncbi.nlm.nih.gov/Blast.cgi). These methods identified 40 candidate genes that were commonly predicted to be possible targets of miR156a (S3 Table). The most highly conserved sequence of a putative binding site among various species is located in the 3’ UTR of JAMA (Fig 3A). The minimum free binding energy was calculated to be –27.3 kcal/mol. First, we evaluated whether miR156a mimic could negatively regulate JAMA expression. As shown in Fig 3B and 3C, the decrease in JAMA protein expression was confirmed by western blotting, whereas JAMA mRNA expression was not affected. Moreover, a luciferase reporter assay was performed to determine whether miR156a had an effect on the 3’ UTR of JAMA. The wild-type or mutant miR156a complementary sites (CS) were cloned into a luciferase reporter vector and transfected into 293T cells combined with miR156a mimic (Fig 3D). As expected, compared with the negative controls, co-transfection of miR156a mimic with the human JAMA 3’ UTR wild type reporter (psiCHECK2-JAMA 3’ UTR-WT) resulted in a highly significant decrease in luciferase activity. A consistent lack of decrease in luciferase activity was observed when miR156a mimic or negative control was co-transfected with the empty vector or the mutant reporter (psiCHECK-JAMA-3’ UTR-MUT), indicating that the predicted site is a direct target of miR156a (Fig 3E).

### JAMA is Essential for the MiR156a Mimic Inhibited EMT of NPC Cells and Involved in the Activation p-Akt

**Fig 4:**
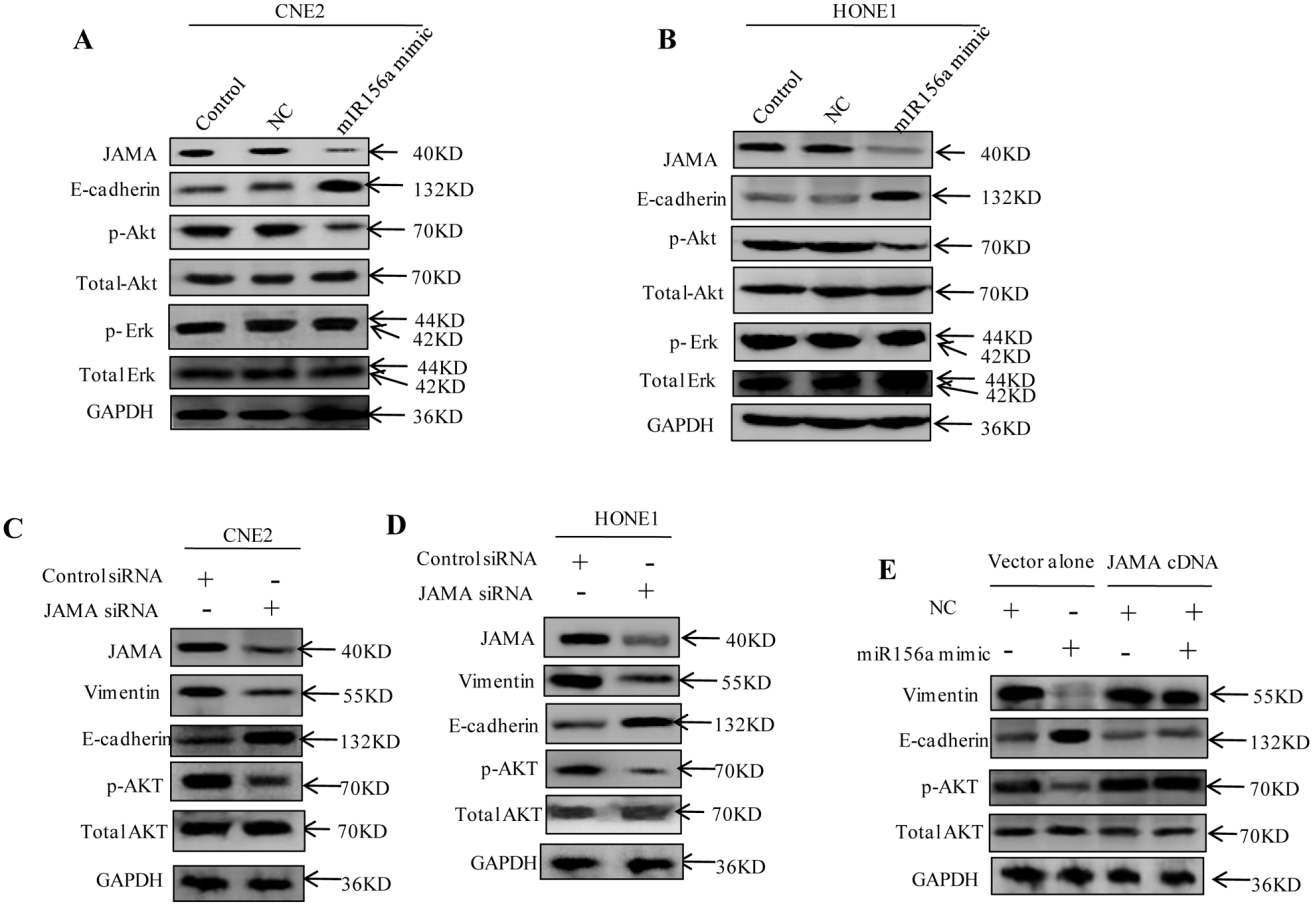
JAMA is essential for miR156a mimic-induced EMT of NPC cells and is involved in the activation of p-Akt *in vitro*. (A and B) Analysis of protein expression by western blotting, JAMA and p-Akt protein is reduced by miR156a mimic. (C) JAMA, vimentin and p-Akt are reduced by small interfering RNA (JAMA-siRNA) in CNE2 (C) and HONE1 cells (D). (E) Western blotting assay analyzed CNE2 cells transfected with the JAMA plasmid or vector control, along with miR156a or negative controls.

Previous studies have confirmed that JAMA regulates epithelial cell proliferation through Akt/β-catenin signaling [16]. Our results consistently indicated that the epithelial marker E-cadherin was significantly increased in CNE2 and HONE1 cell lines, whereas the protein expression of JAMA and p-Akt was reduced after transfection with miR156a mimic. However, the ERK signaling pathway was not affected (Fig 4A and 4B). JAMA was shown to be an authentic target for miR156a, but further investigation was needed to determine whether miR156a mimic inhibited EMT through direct down regulation JAMA. First, we investigated whether reducing JAMA expression might exert a similar repression effect on miR156a mimic. The results indicated that after transfection of JAMA-siRNA into CNE2 and HONE1 cell lines the expression of E-cadherin was increased, whereas p-Akt and the mesenchymal cell marker vimentin were substantially knocked down (Fig 4C and 4D).

To further test whether JAMA is a direct functional mediator of the EMT of NPC cells inhibited by the miR156a mimic, a pWPI-JAMA overexpressed vector was successfully constructed. Then, CNE2 cells were transfected with the JAMA plasmid or vector control, along with miR156a mimic or negative control. Western blotting indicated that JAMA could partially abrogate the miR156a mimic-repressed EMT in NPC cells by activating p-Akt (Fig 4E). These results strongly support that miR156a mimic regulates the mesenchymal phenotype that directly targets the 3’ UTR of JAMA.

## Discussion

miRNAs have emerged as important regulators of a wide range of plant physiologic processes and have contributed to the metastasis of most human cancers [1,17]. With the development of high-throughput sequencing technology, the discovery of plant miRNAs has increased dramatically [4]. Although broccoli possesses anticancer properties, no miRNAs have been reported in broccoli [5]. EMT plays a pivotal role in the progression and metastasis of malignant tumors [11]. We therefore hypothesized that miRNAs from broccoli might have anticancer effects. Our results indicate that there are 84 conserved miRNAs and 184 putative novel miRNAs in broccoli found by sequencing technology. Among these miR156a was expressed the most. In addition, synthetic miR156a mimic inhibited the EMT of NPC cells *in vitro*. Furthermore, it was confirmed that JAMA was the target of miR156a, as validated by 3’ UTR luciferase reporter assays and Western blotting analysis. Knockdown of JAMA was consistent with the effects of 156a mimic on the EMT of NPC, and the upregulation of JAMA could partially restore EMT repressed by miR156a mimic. Taken together, these miRNA profiles of broccoli provide a fundamental basis for further research. Moreover, the discovery of miR156a mimic may have clinical implications for the treatment of patients with NPC.

High-throughput sequencing methods now provide a rapid and efficient additional approach to identifying and profiling populations of small RNAs. Similar to the typical size distribution of small RNAs observed in other plants [18], our results indicate a range of small RNAs, 21–24 nt in length, in broccoli, with most of the unique sequence reads being 24 nt in length. Genomic location analysis also indicated that a majority of small RNAs were almost evenly located in the strand of chromosome. These results were the same with small RNAs from *Phaeodactylum tricornutu*m [19]. Moreover, similar to previous studies, the present study also found that miRNA sequences from broccoli start with uridine (U) [20].

Currently, > 24 521 hairpin sequences and 30 424 mature sequences have been identified in plants, animals, viruses, and even some unicellular organisms (miRBase release 20.0, June, 2013, http://mirbase.org/). Although a large number of miRNAs have been identified, none have been reported in broccoli. The identified conserved miRNAs have been shown in a variety of plant species. For example, miR319, miR156/157, miR169, miR165/166, and miR394 were found in more than 40 plant species [21]. However, miRNA in broccoli displayed a significantly varied abundance from each other. For example, the majority of broccoli miRNAs were sequenced in > 1000 reads, and some rare miRNAs were detected in about 10 reads. Inconsistently, previous studies have confirmed that miR159, miR166a, miR164, miR171f, and miR168 in *Brassica rapa* and *Arabidopsis* had a relatively high number of reads, whereas miR169 family members had a low number of reads [18,22]; miR156a, miR168a, and miR157d topped the list of copy numbers in broccoli. This may suggest a species-specific expression profile for miRNA. Generally, new species-specific miRNAs are considered to be young miRNAs that have recently evolved, and are often expressed at a lower level than conserved miRNAs, as was reported for *Arabidopsis* and *Brassica napus* [18]. This observation is also true for many of the new broccoli miRNAs identified here. Moreover, consistent with previous studies in plant miRNA, most putative miRNA sequences were confirmed to start with uridine (U) as their 5’ first nucleotide, and the largest population was the 21-nt miRNAs [20]. These novel miRNA precursors had fewer negative folding free energies than plant miRNA precursors (–71.0 kcal/mol in rice and –59.5 kcal/mol in *Arabidopsis*) [23].

Previous studies have shown that JAMA induces endothelial cell migration and adhesion [24]. Our previous results also suggested that JAMA plays a role in regulating the EMT of NPC cells [10]. The present study indicated that miR156a mimic inhibited the EMT of NPC cells by targeting JAMA. Based on this and previous evidence, we can conclude that genuine plant miR156a from broccoli, not miR156a mimic, may inhibit the EMT of NPC cells by targeting the 3’ UTR of JAMA. This conclusion is also supported by the observation reported by Zhang *et al*. that plant miR168a specifically targets mammalian low-density lipoprotein receptor adaptor protein 1(LDLRAP1) [6]. Taken together, this confirms that miRNA156a of broccoli may play an important role in regulating the EMT of NPC. Generally, miR156a is conserved in plants. The conserved miR156a have been found in about 40 plant species (miRBase release 20.0). It was confirmed that miR156a play an important role in regulateing flowering and responsing to stress [25]. Xu *et al*. indicated that vegetative phase change in flowering plants is regulated by a decrease in the level of miR156. Most of the miR156 in Arabidopsis thaliana shoots is produced by miR156a and miR156c [26]. Yang *et al*. confirmed that *sugar promoted vegetative phase change in A*. *thaliana by repressing the expression of miR156a and miR156c* [27]. In the present study, we indicated that miRNA156a of broccoli may play an important role in regulating the EMT of NPC. However, we have no idea whether miR156a in other plants may also have the same effects. In our future study, we will pay more attention to the effect of miR156a from other plants.

As we know, plants and animals share several similarities in the basic phenomenon underlying miRNA biogenesis and its functionality. However, many differences are as follows. First, significant numbers of animal miRNAs are located in the introns of pre-mRNAs, though both plant and animal miRNA genes are predominantly located in what is conventionally termed the intergenic regions. Moreover, polycistronic pri-miRNA transcripts with multiple miRNA-generating hairpins have been detected but are rare in plants [28]. Second, there are many differences in miRNA biogenesis. Most notably, the Drosha gene that processes the pri-miRNA to the pre-miRNA in animals in the cytoplasm is absent from plant genomes; this function is carried out by the plant RNase-III-like protein, Dicer-like 1 (DCL1). DCL1 appears to catalyze the processing of the primary miRNA transcript to form the miRNA:miRNA* duplex in the nucleus. More subtle differences include somewhat more pairing between the miRNA and the other arm of the stem loop in plants compared to animals, a tighter distribution of plant miRNA lengths that centers on 21 nt rather than the 22- to 23-nt lengths most often seen in animals and perhaps a stronger preference for a U at the 5’ terminus of the plant miRNAs [29]. Third, animal miRNAs typically anneal with imperfect sequence complementarity to the 3’UTR of the target mRNAs and reduce protein levels through miRNA-mediated transational repression [30]. In contrast with animal miRNAs, most plant miRNAs cause mRNA degradation by interacting with their target sequence through prefect or near-perfect complementarity, usually in the protein coding region [31]. Interestingly, Zhang *et al*. [6] showed that plant miRNA168a prevented mRNA from being translated by binding to exons on the target genes. However, our data show that miR156a mimic bound to its binding site located in 3’ UTR of the JAMA gene and then reduced the level of JAMA protein expression, but did not affect the mRNA level. These results suggest that miRNA156a mimic executes its function similarly to mammalian miRNA.

A recent publication suggests that some plant miRNAs can pass through the mouse GI tract and enter the circulatory system in association with microvesicles. Furthermore, they demonstrated that plant miRNAs in food can regulate the expression of target genes in mammals [6]. However, zhang *et al*. compared some of the most abundant plant miRNAs in public animal sRNA datasets against the NCBI nucleotide sequence database (NT) and conducted insect feeding studies. Their results indicated that the observed plant miRNAs in animal small RNA datasets are not universal in animals [32]. Dickinson *et al*. also indicated that small RNAs from the plasma and liver of mice fed on rice chow did not reveal any measurable uptake of rice grain miRNAs, including osa-miR168a [33]. Unfortunately, we only confirmed that miR156a mimic can target JAMA to repress the EMT of human NPC cells. However, there are differences between miRNA mimics and genuine plant miRNAs: for example, plant miRNAs are 2’-*O*-methyl modified on their terminal nucleotide, whereas animal miRNAs or miRNA mimics synthesized by us with free 2’ and 3’ hydroxyls [6]. Moreover, the present studies were performed *in vitro* using miRNA mimics, although miRNAs were found in mammalian serum in some reports. Therefore, we do not know whether genuine miR156a from broccoli—which may be different from mimics—can regulate the expression of JAMA genes in mammals. Undoubtedly, follow-up studies are urgently required to carefully evaluate the uptake of food-derived miRNAs and the role of the genuine miR156a from broccoli in animals.

In summary, 84 conserved miRNAs and 184 novel miRNAs were found for the first time in broccoli. Moreover, miR156a mimics inhibited the EMT of NPC cells by targeting the 3’ UTR of JAMA. Taken together, these miRNA profiles of broccoli provide a fundamental basis for further research. Moreover, the discovery of miR156a mimics may have clinical implications for the treatment of patients with NPC.

## Supporting Information

S1 Table Sequence of primers for real-time PCR. (DOCX)

S2 Table Sequence of primers for RNAi constructs, constructed cDNA and the 3’ UTR of the JAMA. (DOCX)

S3 Table Potential mammalian genes identified as miR156a targets. (DOCX)

## Abbreviations

JAMA: junctional adhesion molecule-A
EMT: epithelial-mesenchymal transition
NPC: nasopharyngeal carcinoma
PKB(also known as Akt): protein kinase B
FBS: fetal bovine serum
shRNA: short hairpin RNA
PBS: phosphate buffered saline
ERK1/2: extracellular regulated protein kinases
3’UTR: 3’untranslated region
RT-PCR: reverse-transcriptase polymerase chain reaction
