# Supplementary material for: miR156a Mimic Represses the Epithelial–Mesenchymal Transition of Human Nasopharyngeal Cancer Cells by Targeting Junctional Adhesion Molecule A
Source: PLoS One. 2016 Jun 24;11(6):e0157686. doi: 10.1371/journal.pone.0157686 (PMC4920421; doi:10.1371/journal.pone.0157686)
Supplement: S1 Table — (DOCX) [file pone.0157686.s001.docx]

**S1 Table. Sequence of primers for real-time PCR**

| **Name** | **Primer** | **Sequence** |
| --- | --- | --- |
| miR156a | RT primer | GTCGTATCCAGTGCAGGGTCCGAGGTATTCGCACTGGATACGACGTGCTC |
|  | Forward | GCTCGC TGACAGAAGAGAG |
| miR156b | RT primer | GTCGTATCCAGTGCAGGGTCCGAGGTATTCGCACTGGATACGACGTGCTC |
|  | Forward | GCTCGC TTGACAGAAGATAG |
| miR157d | RT primer | GTCGTATCCAGTGCAGGGTCCGAGGTATTCGCACTGGATACGACGTGCTC |
|  | Forward | GATCGC TGACAGAAGATAG |
| miR158a | RT primer | GTCGTATCCAGTGCAGGGTCCGAGGTATTCGCACTGGATACGACTGCTTTG |
|  | Forward | GCTCACTG TTTCCAAATGTAG |
| miR158b-3P | RT primer | GTCGTATCCAGTGCAGGGTCCGAGGTATTCGCACTGGATACGACGCTTTG |
|  | Forward | GCTCACTG TTTCCAAATGTAG |
| miR164a | RT primer | GTCGTATCCAGTGCAGGGTCCGAGGTATTCGCACTGGATACGACTGCACG |
|  | Forward | GATAC TGGAGAAGCAGGGC |
| miR165a-3P | RT primer | GTCGTATCCAGTGCAGGGTCCGAGGTATTCGCACTGGATACGACGGATG |
|  | Forward | GTCACTG TCGGACCAGGC |
| miR167c | RT primer | GTCGTATCCAGTGCAGGGTCCGAGGTATTCGCACTGGATACGACTAGATC |
|  | Forward | GTCATTG TGAAGCTGCCAG |
| miR168a | RT primer | GTCGTATCCAGTGCAGGGTCCGAGGTATTCGCACTGGATACGACTTCCCG |
|  | Forward | GTTAC TCGCTTGGTGCAGG |
| miR172a | RT primer | GTCGTATCCAGTGCAGGGTCCGAGGTATTCGCACTGGATACGACATGCAG |
|  | Forward | GCCAGTGC AGAATCTTGATG |
| miR391 | RT primer | GTCGTATCCAGTGCAGGGTCCGAGGTATTCGCACTGGATACGACTGGCG |
|  | Forward | GTCACTG TTCGCAGGAGAG |
| miR400-3P | RT primer | GTCGTATCCAGTGCAGGGTCCGAGGTATTCGCACTGGATACGACTCCAG |
|  | Forward | GTCACTG GACTTATAATAAGTC |
| miR408-5P | RT primer | GTCGTATCCAGTGCAGGGTCCGAGGTATTCGCACTGGATACGACCATGC |
|  | Forward | GTCCTG ACAGGGAACAAGC |
| miR774b-5P | RT primer | GTCGTATCCAGTGCAGGGTCCGAGGTATTCGCACTGGATACGACGTCTG |
|  | Forward | GTCGATG TGAGATGAAAGATC |
| Universal reverse | | TGCAGGGTCCGAGGTATTC |
| U6 | Forward | GAACGATACAGAGAAGATTAGC |
|  | Reverse | TTGGACCATTTCTCGATTTGTG |
| JAMA | Forward | CCGTCCTTGTAACCCTGATT |
|  | Reverse | CTCCTTCACTTCGGGCACTA |
| GAPDH | Forward | GGACTCATGACCACAGTCCA |
|  | Reverse | CCAGTAGAGGCAGGGATGAT |
