# Supplementary material for: miR156a Mimic Represses the Epithelial–Mesenchymal Transition of Human Nasopharyngeal Cancer Cells by Targeting Junctional Adhesion Molecule A
Source: PLoS One. 2016 Jun 24;11(6):e0157686. doi: 10.1371/journal.pone.0157686 (PMC4920421; doi:10.1371/journal.pone.0157686)
Supplement: S2 Table — (DOCX) [file pone.0157686.s002.docx]

**S2 Table. Sequence of primers for RNAi constructs, constructed cDNA and the 3’ UTR of the JAMA**

| **Name/ direction** | **Sequence 5′🡪3′** |
| --- | --- |
| Primer sequence for JAMA cDNA | |
| Forward | GGCTTAATTAAATGGGGACAAAGGCGCAAG |
| Reverse | GCGGTTTAAACTCACACCAGGAATGACGAG |
| Primer sequence for 3’UTR of JAMA | |
| Forward | AGCCTCGAGACGACCAGGGCCAGCTGTTCT |
| Reverse | ATCGCGGCCGC GCTCTCCACAACAAGAGCTCCCA |
| JAMA shRNA | |
| #1 | GAGAATACAGCTGTGAGGC |
| #2 | CGUACGCGGAAUACUUCGA |
