# Supplementary material for: miR156a Mimic Represses the Epithelial–Mesenchymal Transition of Human Nasopharyngeal Cancer Cells by Targeting Junctional Adhesion Molecule A
Source: PLoS One. 2016 Jun 24;11(6):e0157686. doi: 10.1371/journal.pone.0157686 (PMC4920421; doi:10.1371/journal.pone.0157686)
Supplement: S3 Table — (DOCX) [file pone.0157686.s003.docx]

**S3 Table. Potential mammalian genes identified as miR156a targets.**
